# Volatile Metabolites of *Piper eriopodon* (Miq.) C.DC. from Northern Region of Colombia and Assessment of In Vitro Bioactivities of the Leaf Essential Oil

**DOI:** 10.3390/molecules28062594

**Published:** 2023-03-13

**Authors:** Amner Muñoz-Acevedo, María C. González, Yurina Sh. De Moya, Juan D. Rodríguez

**Affiliations:** 1 Departamento de Química y Biología, Universidad del Norte, Barranquilla P.O. Box 1569, Colombia; 2 Facultad de Ciencias Básicas y Biomédicas, Universidad Simón Bolívar, Barranquilla P.O. Box 50595, Colombia

**Keywords:** *Piper eriopodon*, gibbilimbol B, volatile fractions/essential oil, antibacterial effects, antiradical capacity, cytotoxicity/anticancer, repellency/AChE

## Abstract

*Piper eriopodon* is one of the *Piper* species found in the Sierra Nevada de Santa Marta, and the species has been reported with different compositions of their essential oils (EO). In this study, the volatile fractions/essential oil (by HS-SPME/SDE/MWHD-GC–MS/^1^H-NMR) of different parts from the plant were characterized, and assessments of the in vitro bio-properties of the leaf EO were conducted. The results indicated the following: (i) in the volatile fractions were β-caryophyllene (~23%)/myrcene (~20%) (inflorescences) and β-caryophyllene (~43%)/β-selinene (~20%) (leaves) using HS-SPME; myrcene (~31%)/β-pinene (~23%) (inflorescences), gibbilimbol B (~60%) (fruits) and gibbilimbol B (~46%)/β-caryophyllene (~11%) (leaves) through SDE; (ii) leaf EO contained gibbilimbol B (~72%), confirmed with ^1^H-NMR; (iii) the cytotoxic values (µg/mL) in erythrocytes/lymphocytes/Hep-2 were HC_50_: 115 ± 3 (eryth.), LC_50_: 71 ± 4 (lymph.) and LC_50_: 33 ± 2 (cell-line); (iv) the antibacterial susceptibilities (ϕ inh. zone, mm; 4–16 µg EO) were 22.5 ± 0.4–97 ± 4 (*Staphylococcus aureus*), 23 ± 2–77 ± 4 (*Escherichia coli*) and 17 ± 1–48 ± 3 (*Listeria monocytogenes*); (v) the TAA value was 2249 ± 130 mmol Trolox^®^/kg; (vi) the IC_50_ value was 13±1 µg/mL (AChE) with 20 ± 0–37 ± 6% repellency (2–4 h, *Sitophilus zeamais*). Thus, the EO of *P. eriopodon* leaves from northern Colombia could be a promising species for sustainable exploitation in the future due to its outstanding bioactivities.

## 1. Introduction

Colombia is the second richest country in the world in plant biodiversity (20,299 species), and registers 604 species of Piperaceae [1,2,3]. From this number, *ca*. 50 *Piper* spp. (four endemic and the others also found/disseminated in the rest of the Caribbean region and the Northern Andes) are distributed in the Sierra Nevada de Santa Marta (northern region of Colombia) [4].

One of them, *Piper eriopodon* (Miq) C.DC. (syn. *Artanthe eriopoda* Miq., *P. leptophyllum* C. DC.; common name: “cordoncillo”) is a shrub (up to 4 m high) with erect inflorescences (length between 7–9 cm) and leaves that are scaly to the touch with a characteristic odor; it is native to Colombia, Venezuela and Ecuador [5,6,7]. However, there is little information in Colombia on traditional uses of the plant; even so, Saavedra Barrera [7] stated that the plant has analgesic, diuretic and antirheumatic properties, and is used as a treatment for kidney stones, bronchial conditions and as an antidote against snake bites. In the northern Colombian region, it is considered a weed.

Notwithstanding this, the medicinal properties of *Piper* species are well known, based on scientific validations/phytochemistry [8,9,10,11]; moreover, some scientific literature consulted on extracts (e.g., hexane, methanol, ethanol, butanol, dichloromethane), essential oils (inflorescences/leaves/stems) and isolated compounds (e.g., gibbilimbol B) of *P. eriopodon* have evidenced that these substances have antibacterial (e.g., *Mycobacterium bovis*, *M. tuberculosis*, *Staphylococcus aureus*), antifungal (e.g., *Aspergillus fumigatus*, *Botrytis cinerea*, *Fusarium solani*, *F. oxysporum*, *Trichophyton mentagrophytes*, *T. rubrum*), herbicidal, anticancer (e.g., A549, HeLa, HepG-2, MDAMB-231, MCF-7, PC-3 cell lines)/cyto-toxic (e.g., *Artemia franciscana*, *human dermal fibroblast*, RAW264.7 macrophages, Vero cell line), nematicidal (*Meloidogyne* spp. and *Radopholus* spp.) and antioxidant (e.g., ABTS^+•^, DPPH^•^, ORAC) properties [12,13,14,15,16,17,18,19,20,21,22,23,24,25,26,27,28,29], as well as chemical variability.

Nevertheless, information on the composition of volatile fractions of the plant, as well as the effects of the leaf EO as a repellent/acetylcholinesterase inhibitor, antibacterial (*Listeria monocytogenes*) and cytotoxin on erythrocytes/lymphocytes/Hep-2 cell line were not found in the science literature consulted, even though some reports ([7,12,13,14,20,29]) on the chemical composition [dill apiole (~39%) and eucalyptol (~37%)] of the leaf/(stem) EO and its antimicrobial/cytotoxic/antioxidant effectiveness (*S. aureus*, *A. fumigatus*, *T. mentagrophytes*, *A. franciscana*, Vero cell line, ABTS^+•^/DPPH^•^) were available, as was previously mentioned. Therefore, the focus of this research was to chemically characterize via GC–MS (gas chromatography–mass spectrometry) the volatile fractions [by headspace solid-phase microextraction/simultaneous distillation-extraction (HS-SPME/SDE)-inflorescences (fruits)/leaves] and essential oil [by microwave-assisted hydrodistillation (MWHD)-leaves] of *P. eriopodon* from the northern region of Colombia, as well as establish the chemical profile of the leaf essential oil (EO) by GC–MS/^1^H-NMR (hydrogen-1 nuclear magnetic resonance). Moreover, as an additional value, the in vitro biological prospecting of this EO was assessed according to cytotoxic (on human cell models), antibacterial (three strains), antiradical (ABTS^+•^) and repellency/AChE inhibition assays.

## 2. Results

### 2.1. Identity of Plant

The botanical sample was identified as *Piper eriopodon* (Miq.) C.DC., and the leaf EO was a yellowish liquid (at room temperature) which became solid at 4 °C; the EO yield was 0.08%.

### 2.2. Chemical Composition of the Volatile Metabolites

The constituents (>0.4%) that were positively identified (~91–100%) as volatile metabolites in the different parts of plant, based on the elution order of the total ion current (TIC), are included in Table 1. According to this table, in the volatile fractions through HS-SPME were found the following: (i) β-caryophyllene (~23%), myrcene (~20%) and β-pinene/α-pinene (~19%)/(~10%) in the inflorescences; and (ii) β-caryophyllene (~43%) and β-selinene (~20%) in the leaves. In this manner, monoterpenes (~65%) and sesquiterpenes (~90%) were the principal components of the inflorescences and leaves, correspondingly. Furthermore, the chemical compositions of the SDE extracts were as follows: (i) inflorescence-myrcene (~31%), β-pinene (~23%), α-pinene/gibbilimbol B (each ~14%); (ii) fruit-gibbilimbol B (~60%), β-pinene (~10%)/myrcene (~9%); and (iii) leaf-gibbilimbol B (~46%), β-caryophyllene (~11%), β-pinene (~9%)/myrcene (~7%). When considering composition based on compound families, the inflorescences were composed of monoterpenes (~74%) and simple phenols (~14%); the fruits were made up of simple phenols (~60%) and monoterpenes (~30%); and the leaves consisted of simple phenols (~46%), monoterpenes (~31%) and sesquiterpenes (~19%). Finally, the leaf EO comprised gibbilimbol B (~72%) and β-caryophyllene (~9%); the representative families of the EO were simple phenols (~72%) and sesquiterpenoids (~22%).

As an additional process to corroborate the presence of gibbilimbol B and other terpene constituents in the *P. eriopodon* leaves and inflorescences, ethyl acetate extracts were obtained from these parts and analyzed using GC–MS; the chemical compositions are included in Table 1. Accordingly, gibbilimbol B (~70%) and β-caryophyllene (~7%) were the majority constituents found in the inflorescence extract (composition similar to leaf EO), while β-caryophyllene (~19%), phytol (~11%) and gibbilimbol B/β-selinene (~10% each) were for the leaf extract.

**Scheme 1:**
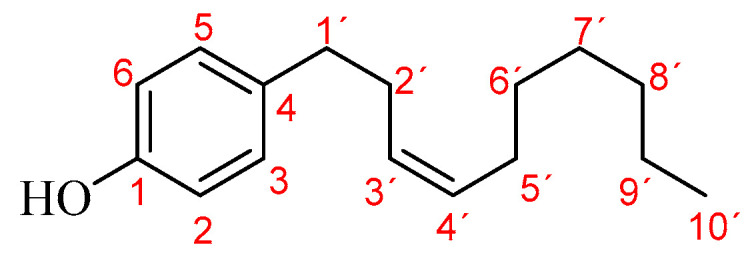
Gibbilimbol B.

^1^H-NMR was used for obtaining the characteristic profile of EO and confirming the presence of gibbilimbol B (main constituent, >70%—Scheme 1). The signals recorded in the profile EO are presented below.

The characteristic signals for the yellowish liquid (EO) via ^1^H-RMN (CDCl_3_, 400 MHz) were as follows: δ 7.03 (d), 6.74 (d), 5.88 (s), 5.46–5.37 (m), 5.30 (s), 5.10–4.82 (m), 4.01 (s), 3.75 (s), 3.64 (t), 3.30 (d), 2.89 (dd), 2.59 (t), 2.35–2.31 (m), 2.29–2.20 (m), 2.17–2.03 (m), 2.00–1.92 (m), 1.82–1.43 (m), 1.36–1.20 (m), 1.12–1.03 (m), 1.00–0.96 (m), 0.88 (t), 0.84–0.77 (m), 0.72 (s).

From previous signals, those corresponding to gibbilimbol B [C_16_H_24_O, GC–MS (EI, 70 eV)-t_R_ 80.62 min, m/z (%): 232.18 (M^+•^, 6), 107.04 (100)] were as follows: ^1^H-δ 7.03 (2H_3,5_, d, J = 8.4 Hz, 3 and 5-H_Ar_), 6.74 (2H_2,6_, d, J = 8.4 Hz, 2 and 6-H_Ar_), 5.46-5.37 (2H_3′,4′_, m, –HC=CH–), 2.59 (2H_1′_, t, J = 7.2 Hz, C_Ar_–CH_2_–), 2.29-2.20 (2H_2′_, m, –CH_2_–CH=C–), 2.00-1.92 (2H_5′_, m, –C=CH–CH_2_–), 1.36-1.23 (8H_6′–9′_, m, –(CH_2_)_4_–) and 0.88 (3H_10′_, t, J = 6.6 Hz, –CH_3_). In addition, some unique/particular signals assigned to hydrogen atoms bonded to sp^2^ carbons of β-caryophyllene [C_15_H_24_, GC–MS (EI, 70 eV)-t_R_ 47.92 min, m/z (%): 204.18 (M^+•^, 4), 93.09 (100)] were observed; thus, δ 5.10-4.82 (H, m, –HC=C–), 4.01 (H_a_, s, –C=CH_2ab_) and 3.75 (H_b_, s, –C=CH_2ab_).

Otherwise, considering the biological prospects of the EO, the results obtained were reported in Table 2. According to the table, the cytotoxicity on erythrocytes, reported as the 50% hemolytic concentration (CH_50_-as a measure (approximation) of the skin-irritant capacity of a substance), established that the EO was moderately hemolytic (100 µg/mL < HC_50_ < 1000 µg/mL); whereas, the EO was cytotoxic (LC_50_ < 100 µg/mL) against lymphocytes/Hep-2 line, being more effective on the cell line than on lymphocytes, and showing a selectivity index of 2.2 on the Hep-2 cells. As per the F test, there were significant differences between each of the control substances and the EO for the tested cells (*p* ≤ 0.0001), as well as amongst the cell types.

**Figure 1:**
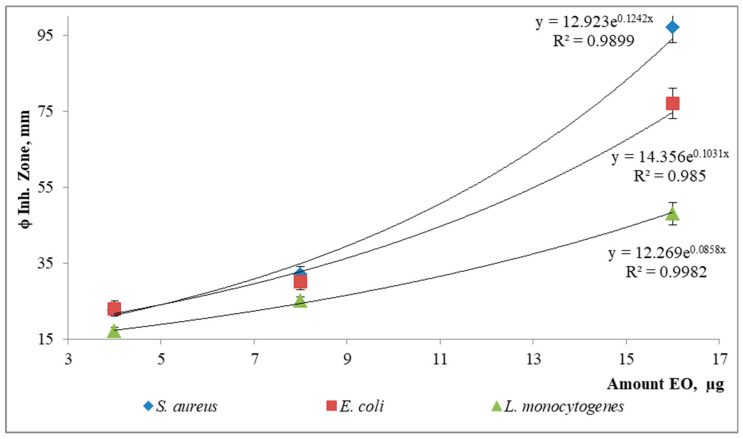
Effect of the EO amount on the inhibition of bacterial growth.

A remarkable bioproperty demonstrated by *P. eriopodon* EO was its high efficacy for inhibiting bacterial growth. Thereby, the three tested strains (*S. aureus*, *E. coli* and *L. monocytogenes*) were susceptible to the different evaluated amounts (4–16 µg) of the EO. The effect of the tested amount (µg) of EO on the inhibition of radial growth for each bacterial species is shown in Figure 1, and from this, it could be observed that the effect on the inhibition of bacterial growth (for all of them) was dose dependent in an exponential mode. It is worth highlighting that the lowest amount (4 µg) of tested EO was capable of inhibiting equal to or higher than the positive control (ϕ inh. zone of EO ≥ ϕ inh. zone of control antibiotic). Nonetheless, the descending order of bacterial susceptibility (from highest to lowest) towards the EO was *S. aureus* > *E. coli* > *L. monocytogenes*. The Anova results for these data showed that the general effect of EO on the evaluated bacterial strains was similar [there were no significant differences, [*p*: 0.2357, F (2.1194) < F_crit_ (6.9443)], whilst the effect of the different amounts of EO was significant [*p*: 0.01, F (16.053) > F_crit_ (6.9443)] on each strain, all of the above evidenced by the trends shown in Figure 1. In turn, the same Anova demonstrated that the lowest amount (4 µg) of the EO evaluated on strains had no differences [*p*: 0.1468-0.2394, F (3.18–5.35) < F_crit_ (18.5–19)] with the control antibiotic (4–30 µg) when compared, indicating that the EO and control had similar antibacterial effectiveness under the conditions of this assay. In contrast, the other EO amounts tested (8–16 µg), pursuant to the Anova, were significantly different (*p*: 0.002–0.02, F (52.88) > F_crit_ (18.5)]) in relation to the standard antibiotic.

Then, the repellent effect against *Sitophilus zeamais* (Coleoptera: Curculionidae) and the in vitro inhibition of the acetylcholinesterase enzyme (AChE) were also evaluated for this EO. As a result, the degree of repellency for the EO was moderate (~1.6–2.9 ratios, compared to and favoring the “control” standard), and the inhibitory effect of EO on AChE was significant (*p*: 0.0004, F: 0.0004), although it did not exceed the value obtained for the positive control.

Finally, according to the TAA value (2249 ± 130 mmol Trolox^®^/kg) of the EO evaluated, the reactivity of the EO towards the ABTS^+•^ radical-cation, as a measure of its antioxidant capacity, was slightly higher (ratio 1.04) than that of the “control” antioxidant (BHA); however, there was not a significant difference (*p*_F_: 0.1902, *p*_t-s_:0.07/0.140, F: 0.235) between these values, and therefore, the radical-scavenging capacity of *P. eriopodon* EO was comparable to the BHA.

## 3. Discussion

As a starting point for the discussion, the EO yield regarding the consulted literature showed significant differences; e.g., Castañeda [12] and Ustáriz Fajardo et al. [14] reported EO yields of 0.16% and 0.19%, respectively, values that were equal to or greater than double when compared with that of this research (0.08%). Nonetheless, Ustáriz Fajardo et al. isolated the EO from leaves/stems of the plant, while the other authors obtained it from the leaves. Even with this fact, the differences could also be attributed to some environmental factors such as climate conditions (rainy or dry season-drought stress), soil type (organic matter and mineral contents) and location (latitude, longitude, relative moisture) where the plant was collected, as reported by Fernández-Sestelo and Carrillo [30], García-Caparrós et al. [31] and Şanli and Karadoğan [32].

If the volatile chemical characterization is considered, the composition of volatile fractions/essential oil obtained in this paper with those found in the scientific literature showed similarities in some cases and variations in others. In the same way, no record on the composition of the volatile fractions of the different parts of *P. eriopodon* was available; therefore, studies on the volatile fractions of other *Piper* species were used. Thus, Hao et al. [33] reported that the volatile fractions determined in the leaves of 10 *Piper* spp. (*P. betle*, *P. auritum*, *P. retrofractum*, *P. hainanense*, *P. pseudofuligineum*, *P. laetispicum*, *P. flaviflorum*, *P. cathayanum*, *P. puberulum*, *P. longum*), using HS-SPME [with DVB/CAR/PDMS (50/30 μm) as the fiber coating] and GC/MS, contained terpene hydrocarbons between ~20-86%, and in eight (*P. betle*, *P. retrofractum*, *P. hainanense*, *P. pseudofuligineum*, *P. laetispicum*, *P. cathayanum*, *P. puberulum*, *P. longum*) of ten species, this type of compound family was predominant (~47–86%). The main terpenes identified were β-caryophyllene (8.5 ± 0.2-27.70 ± 0.08%), myrcene (16.06 ± 0.04–34.9 ± 0.4%) and ocimene (15.6 ± 0.4–31.5 ± 0.6%). Furthermore, Liu et al. [34] and Jirovetz et al. [35] obtained similar results when they studied *P. nigrum*/*P. longum* and *P. nigrum*/*P. guineense*, in turn. For these species, the volatile fractions were composed of mono-/sesquiterpenes, and with β-caryophyllene (~24%/~33% and ~52%, respectively) as the main constituent for three of four species. These data were similar to those included in this study (family of terpenes), regardless of whether the fiber coating used was different (PDMS-100 µm). Possibly, volatiles recognized in the inflorescences/leaves [β-caryophyllene (~23–43%) and myrcene (~20%)] of the plant could be playing an ecological role (e.g., chemical messenger of alert (herbivores, as aphids), pollinator attractants [36,37,38,39,40,41]).

When the compositions of the volatile fractions obtained by the techniques used (SPME and SDE) were compared, they differed; that is, monoterpenes (~5–65%)/sesquiterpenes (~34–90%) were the predominant constituents in the inflorescences/leaves according to HS-SPME, whilst simple phenols (~14–60%)/monoterpenes (~30–74%)/sesquiterpenes (~9–19%) were present in the fruits/leaves/inflorescences via SDE. These differences could be related to the pre-established parameters in each method per se; e.g., the extraction temperatures and times for SPME were 50 °C and 30 min, whereas for SDE, 100 °C (in the reservoir of the plant) and 2 h were used; furthermore, due to the chemical nature of the extractions, CH_2_Cl_2_ was used in SDE and a PDMS fiber (non-polar) in SPME. Thus, applied SDE/SPME techniques provided complementary information on the chemical profiles of volatile fractions of the different parts from *P. eriopodon*, which would be in agreement with Kung et al. [42].

Likewise, the chemical compositions of the SDE extract (volatile fraction) and the EO of leaves were similar, with certain differences in the relative amounts. However, the EO composition was different from those reported by Tangarife-Castaño et al. [13], Castañeda et al. [29], Uztáris-Fajardo et al. [14] and Velandia et al. [20], whose EOs were composed of dillapiole (~39%)/β-caryophyllene (~8%), 1,8-cineole (~37%)/β-pinene (~9%) and α-pinene (~19%)/β-pinene (~16%)/β-caryophyllene (~12%)/caryophyllene oxide (~11%), respectively. It is important to mention that Muñoz [17] identified gibbilimbol B (~86%) and apiole (~4%) as the main components of the *P. eriopodon* EO, but from fruits.

On the other hand, the ^1^H-NMR signals of the leaf EO were contrasted with those of gibbilimbol B and β-caryophyllene, according to the literature reports [43,44,45,46]; consequently, both the signals and their multiplicities together with the coupling constants (J) of all the H-atoms coincided (same multiplicities, signals and J), thus confirming the presence of these two compounds in EO.

If the results of the cell bioassays on the leaf EO are taken into account, data on cytotoxicity of EO/extracts from *P. eriopodon* have shown a notable cytotoxic effect according to that described by Velandia et al. [20] on HEK293 (IC_50_: 153 ± 10 µg/mL), MCF-7/HeLa (IC_50_: 50 µg/mL for each), and HepG-2 cells (IC_50_: 140 ± 24 µg/mL) by EO (composed by α-pinene/β-pinene/β-caryophyllene/caryophyllene oxide), as well by Muñoz et al. [16] on A549 (IC_50_: 18 ± 2–34 ± 4 µg/mL), PC-3 (IC_50_: 11.9 ± 0.7–45 ± 4 µg/mL) and MDAMB-231 lines (IC_50_: 21 ± 1–53 ± 4 µg/mL) using EtOH extracts (inflorescences/leaves/wood containing significant amount of gibbilimbol B, compound presumably responsible for cancer-fighting properties). This isolated alkenylphenol evaluated on the cell lines produced IC_50_ values of ~11 µg/mL (on MDAMB-231 line), ~12–17 µg/mL (on MCF-7 cells), ~17 µg/mL (on U373 cells) [16,26,27], ~32 µg/mL (on PC-3 cells) and ~40 µg/mL (on A549 line). The other cell line (KB) presented an ED_50_ value of ~4 µg/mL [43]. Lastly, Tangerife-Castaño et al. [13] determined that the EO (rich in alkenylbenzodioxole and sesquiterpene derivatives) showed an IC_50_ value of 16 ± 1 µg/mL on the Vero cell, which differed significantly from the results on lymphocytes and erythrocyte cells of this research.

The differences found in the LC_50_ and CH_50_ values for lymphocytes and erythrocytes could be related to the particular structure of these cells, as well as their physiological functions; i.e., erythrocyte has primarily a lipid bilayer (plasma membrane) and few organelles; if this cell is (or is not) sensitive to a xenobiotic, its membrane would suffer greater (or less) damage (morphological abnormalities), causing (or not) cell disruption (hemoglobin release-hemolysis) [47]. While the lymphocyte (a more specialized and complete cell type) is more sensitive to changes at the intracellular level; when the cell is exposed to a xenobiotic, and it affects (or not) some vital function or damages (or not) some organelle, cell viability will decrease (or not) [48]. In accordance with the foregoing, possibly the EO would be causing significant damage inside the lymphocyte, and for this reason, the highest cell mortality (low LC_50_) occurred compared to cell disruption in erythrocytes.

Furthermore, the obtained antibacterial results were compared with those reported by Ustáriz-Fajardo et al. [14], Guzman et al. [15] and Orjala et al. [43]. Hence, in the case of Venezuelan EO [14], it was not active against *E. coli* and *Klebsiella pneumoniae*, and moderate on *S. aureus* (MIC 2500 µg/mL). Meanwhile, Guzman et al. obtained MIC values of 25 µg/mL and 128 µg/mL against *M. bovis* and *M. tuberculosis*, respectively, by gibbilimbol B isolated from the EtOH extract of plant leaves. The same authors stated that the phenol significantly inhibited the growth of *S. aureus* and was inactive against *E. coli* and *Pseudomonas putida*; in addition, Orjala et al. listed MIC values for gibbilimbol B on *S. epidermidis* and *Bacillus cereus* of 2 µg/mL and 4 µg/mL, in that order. In spite of this, what was previously described differed from the antibacterial results found in this study, due to (i) the chemical composition of EO (from Venezuela) being different and therefore, its antibacterial effect; (ii) as reported by Guzman et al. and Orjala et al., these authors evaluated the isolated phenol (and not the EO) against the bacterial strains. However, gibbilimbol B (the main constituent of EO from northern Colombia) would be possibly responsible for the notable antibacterial power revealed by the *P. eriopodon* EO.

No less important were the results of the repellency and inhibition of the acetylcholinesterase enzyme activities of the EO, which are discussed below. Thus, based on Jaramillo et al. [49], the degree of repellency of *Piper* spp. EOs could vary according to their chemical compositions. These authors stated that the EOs of *P. aduncum*, *P. dilatatum*, *P. divaricatum* and *P. santifelicis* exhibited percentage repellencies of 99%, 82%, 76% and 33%, respectively, against *Tribolium castaneum* Herbst (coleopteran species related to *S. zeamais*). These EOs constituted dillapiole (48%)/eucalyptol (11%) (*P. aduncum*), apiole (89%) (*P. dilatatum*), eugenol (38%)/metil eugenol (36%) (*P. divaricatum*) and δ-3-carene (35%)/limonene (27%) (*P. sanctifelicis*). Meanwhile, Xiang et al. [50] described that some EOs from *Piper* spp. (*P. hispidimervium*, *P. puberulum*, *P. betle*, *P. austrosinense* and *P. flaviflorum*) displayed a moderate effectiveness to inhibit AChE, with IC_50_ values (mg/mL) of 1.51 ± 0.05, 4.5 ± 0.4, 12 ± 0.1, 14.00 ± 0.01 and 14 ± 2, in turn. All the above values were higher (mg/mL) compared to the one determined in this report (µg/mL); therefore, the EO from *P. eriopodon* is a promising AChE inhibitor agent.

Lastly, the anti-radical capacities (by DPPH^•^ and ABTS^+•^) of some EO and extracts (CH_2_Cl_2_, EtOH, hexane and MeOH) from the plant (leaves and/or stems) were determined by Castañeda Muñoz [12], Correa Navarro et al. [24] and Mesa-Vanegas et al. [28]. Thus, the CH_2_Cl_2_/hexane/MeOH extracts (250 µg) presented values between ~16 ± 4-~17 ± 3 µmol Trolox^®^/g sample by DPPH^•^ [24], whereas the IC_50_ values of the EtOH extracts (containing gibbilimbol B) from the plant leaves/stems were 366 ± 2 µg/mL (leaves)/946 ± 2 (stems) by DPPH^•^ and 282 ± 5 µg/mL (leaves)/>1000 (stems) by ABTS^+•^ [28]. Additionally, the EO (from Cesar, Colombia) presented a low radical-scavenging capacity by ABTS^+•^, with a TAA value of 0.000585 ± 0.000002 mmol Trolox^®^/kg [12], which differed from the value determined (2249 ± 130 mmol Trolox^®^/kg) in this study. The similarity in the reactivity shown by the EO towards ABTS^+•^ (compared to the control antioxidant) could be correlated with the presence of compounds capable of donating electrons and protons simultaneously, and generating species with charge delocalization/stabilization capacity as phenols, e.g., gibbilimbol B (Prior et al. [51], Huang et al. [52], Sánchez-Moreno [53]).

## 4. Materials and Methods

*Plant material.* Samples of fresh leaves/inflorescences (or fruits) from *Piper eriopodon* were collected from the sidewalk “Mundo Nuevo”, Bonda village in the city of Santa Marta (Departamento de Magdalena) in November 2015/May 2016. The location coordinates were longitude: 74°06′00.81″ O and latitude: 11°09′58.86″ N. Taxonomic identification (No. Voucher COL588905) was carried out by the Instituto de Ciencias Naturales at the Universidad Nacional de Colombia. The plant collection was made under Resolution No. 739 of 8 July 2014, conferred by the Agencia Nacional de Licencias Ambientales (ANLA).

*Volatile fractions*. The fractions from different parts (inflorescences/fruits and leaves) of the plants were obtained by two methods: simultaneous distillation-solvent extraction (SDE) according to the methodology described by Godefroot et al. [54], using CH_2_Cl_2_ (2 mL) as solvent; and headspace solid phase micro-extraction (HS-SPME) based on the procedure reported by Muñoz-Acevedo et al. [55], using PDMS (100 µm)-coated fiber. All extracts (SDE) and trapped volatiles (in fiber) were analyzed with GC–MS.

*Isolation of essential oil.* Essential oil was obtained from fresh leaves using microwave radiation-assisted hydrodistillation with a Clevenger-type apparatus, a Dean–Stark reservoir and a modified microwave oven [for home, at 700 W, during 1 h (one cycle/15 min)]. Once the EO was decanted and dehydrated, it was prepared for the spectroscopic analysis (GC–MS and ^1^H-NMR) [56].

*Simple maceration process (SMP).* Total extracts of ethyl acetate (ACS reagent grade) from *P. eriopodon* inflorescences/leaves were obtained via simple maceration. The plant part (0.5–1 g) was sunk in the solvent (5 mL) for seven days at 25 °C (under stirring). The extracts were concentrated (up to 1 mL) and analyzed using GC–MS.

*Analysis by GC–MS.* For the analysis of the volatile metabolites, a Trace 1310 GC coupled to an ISQ Series MS (Thermo Fisher Scientific), with split/splitless inlet (10:1 split ratio), liquid autosampler (AI/AS 1310 Series, Thermo Fisher Scientific)/manual injection (SPME) were used. As well, a column Rxi^®^-1ms (30 m × 0.25 mm ID × 0.5 µm df, Restek Co., Bellefonte, PA, U.S.) was suitable for the chromatographic separation. Temperature programming of the GC oven was performed according to Muñoz Acevedo et al. [55,56]. Chromatographic and spectroscopic data were processed using Thermo Xcalibur^TM^ (Version 2.2 SP1.48) along with AMDIS (Build 130.53, Version 2.70) software.

Linear retention indices were calculated using C_7_-C_35_ aliphatic hydrocarbons and analyzed in the same conditions. The chemical constituents were recognized/identified by comparing their mass spectra together with the linear retention indices with those of the available databases (NIST11, NIST Retention Index and Wiley9) and the consulted/existing literature [57,58,59].

*Analysis by NMR.* The NMR spectrum of hydrogen (^1^H) was acquired to 400 MHz, in an Avance-400 Bruker spectrometer. Chemical shifts were reported in ppm using TMS as the internal reference (δ scale), and CDCl_3_ was used as the solvent and internal standard (^1^H: δ 7.26 ppm). The coupling constants (J) were expressed in Hz.

In vitro biological properties. All assays were carried out 3–5 times, including the positive/negative controls, as well as the suitable statistical treatment of the data.

Cytotoxic, acetylcholinesterase enzyme inhibition and repellent capabilities. The cytotoxicity (on human erythrocytes and lymphocytes, and Hep-2 cell line) along with acetylcholinesterase enzyme inhibition assays were carried out based on the methodology described by Muñoz-Acevedo et al. [60]. The repellent test was carried out based on the preferred area procedure reported by Tapondjou et al. [61].

Antibacterial effects. The bacterial susceptibility to essential oil from *P. eriopodon* leaves was evaluated on *Staphylococcus aureus* (ATCC-25923), *Escherichia coli* (ATCC-25922) and *Listeria monocytogenes* (ATCC-7644) strains using the disk diffusion method as reported by Hudzicki [62].

ABTS^+•^ radical-cation scavenging capacity. The assessment of the antioxidant capacity equivalent to Trolox^®^, expressed as total antioxidant capacity [TAA, mmol Trolox^®^/kg SE (substance evaluated: EO or “control” antioxidant)] was carried out following the procedure described by Muñoz-Acevedo et al. [63].

Statistical treatment. The data obtained from the biological tests of the EO were treated with the corresponding figures of merit (average, standard deviation and relative standard deviation). In addition, the analysis tools used to determine the significance between the data were F-test two-sample for variance (*p* < 0.05, F < 0.05), the t-test (paired two samples for means (*p* < 0.05, F < 0.05)) and two-factor analysis of variance (Anova) (*p* < 0.05, F > F_crit_) in Statistica software (version 10, StatSoft, Inc., Tulsa, OK, USA).

## 5. Conclusions

From this research, the following could be concluded: (i) This is the first report of volatile fractions of the different parts of *P. eriopodon* from Colombia that could be related to some ecological role (defense or chemical messenger) in agreement to the established chemical compositions; (ii) based on the antibacterial efficacy of EO, it could be used as a food protectant/preservative, an attribute that would be reinforced by its anti-radical (antioxidant) capacity equivalent to BHA (synthetic antioxidant used in the industry [64]), which could be replaced by this EO as a natural antioxidant; as well as an antiseptic considering that it could be moderately irritating (as per its CH_50_ value). In addition, the selective cytotoxicity (SI: ~2) of the EO on the Hep-2 line regarding lymphocytes would suggest its probable use as a chemotherapeutic agent (anticancer/anti-proliferative) against cervical adenocarcinoma. Lastly, despite the moderate degree of repellency and significant inhibition of AChE by EO, it could be applied as a bio-pesticide. (iii) As the main component found in the leaf EO was gibbilimbol B (~72% and probably responsible for the bioproperties), the final use/application could be carried out with the mixture (EO matrix) more easily than with the isolated alkenyl-phenol because the technique used (MWHD) would be much faster (1 h) and less tedious than the process with solvents (extraction/concentration/purification).

## Figures and Tables

**Table 1:** Chemical composition of volatile fractions (HS-SPME/SDE), essential oil (MWHD) and ethyl acetate extracts (EA-E) of different parts from *Piper eriopodon*.

No. Peak	Compounds	Retention Index		Relative Amounts, %							
				HS-SPME		SDE			EO	EA-E	
		Calc.	Lit.	INF	L	INF	FR	L	L	INF	L
1	α-Pinene	933	930	10.3	1.9	13.6	4.6	6.4	----	1.6	----
2	β-Pinene	969	970	19.2	2.4	23.3	10.0	8.9	tr	3.6	tr
3	Myrcene	984	981	19.6	tr	31.0	9.3	6.8	tr	1.9	----
4	δ-3-Carene	1004	1005	3.5	0.6	----	1.2	2.0	tr	tr	----
5	α-Tolualdehyde	1006	1011	----	----	1.2	tr	tr	----	----	----
6	*p*-Cymene	1010	1011	tr	----	0.5	tr	tr	----	tr	----
7	β-Phellandrene	1017	1023	0.5	----	1.5	tr	0.4	----		----
8	Limonene	1019	1020	1.5	tr	3.5	0.9	1.1	0.1	0.6	----
9	(Z)-β-Ocimene	1028	1032	7.8	----	0.7	2.6	3.9	2.0	0.9	tr
10	(E)-β-Ocimene	1038	1036	1.4	----	----	0.4	0.5	0.5	tr	tr
11	(E)-Hex-2-enoic acid	1040	1042	----	----	----	----	----	----	----	4.8
12	α-Copaene	1368	1376	3.3	4.2	2.6	1.0	0.4	0.4	1.3	3.5
13	β-Caryophyllene	1407	1421	22.8	42.6	6.5	5.7	10.6	8.6	7.0	19.4
14	β-Copaene	1415	1437	0.8	1.4	----	tr	tr	tr	tr	tr
15	Aromadendrene	1429	1439	----	1.1	----		tr	tr	tr	tr
16	α-Humulene	1439	1454	1.5	5.0	0.5	0.4	1.2	1.1	0.5	1.5
17	γ-Muurolene	1462	1471	0.4	1.2	----	tr	tr	tr	tr	tr
18	Selina-4,11-diene	1468	1475	tr	1.2	----	tr	tr	tr	----	----
19	β-Selinene	1470	1483	1.2	20.2	1.0	0.4	3.5	3.4	0.6	9.8
20	α-Selinene	1481	1491	0.8	5.2	----	tr	0.8	0.9	tr	1.5
21	α-Muurolene	1485	1494	0.7	2.2	----	tr	tr	tr	0.4	2.3
22	(E),(E)-α-Farnesene	1492	1498	0.6	----	----	----	----	----	0.4	----
23	γ-Cadinene	1495	1507	----	0.6	----	----	----	----	----	----
24	*trans*-Calamenene	1498	1502	tr	1.2	----	----	tr	tr	----	----
25	7-*epi*-α-Selinene	1501	1511	tr	4.3	----	tr	0.8	0.9	tr	2.5
26	(E)-Nerolidol	1539	1549	----	----	----	tr	0.5	1.0	----	tr
27	Caryophyllene oxide	1551	1558	----	1.8	----	tr	0.6	0.9	----	2.9
28	Dillapiole	1580	1589	----	----	----	----	----	2.2	----	tr
29	τ-Cadinol	1622	1628	----	----	----	----	tr	0.5	----	----
30	Ylangenol *	1625	1666	----	----	----	tr	0.4	1.1	----	----
31	Unidentified compound (M^+•^ 168.07, BP 124.06)	1679	----	----	----	----	----	----	----	----	8.7
32	Gibbilimbol B	1915	1997	0.9	----	14.2	60.1	45.5	71.7	70.0	10.3
33	Palmitic acid	1938	1970	----	----	----	----	----	----	----	2.9
34	Ethyl palmitate	1945	1978	----	1.1	----	----	----	----	0.4	7.7
35	Stearyl alcohol	1982	2066	----	----	----	----	----	0.5	----	----
36	Phytol	1991	2102	----	----	----	----	----	tr	tr	11.3
37	Alkenylphenol (M^+•^ 260.21, BP 107.07)	1996	----	----	----	----	tr	----	tr	2.4	tr
38	Eriopodol A * (M^+•^ 248.18, BP 123.05)	2015	----	----	----	----	----	----	----	1.2	----
39	Ethyl linoleate	2035	2139	----	----	----	----	----	----	----	2.8
40	Ethyl linolenate	2040	2145	----	----	----	----	----	----	----	3.9
41	Ethyl oleate	2045	2150	----	----	----	----	----	----	----	1.7
42	Ethyl stearate	2070	2175	----	----	----	----	----	----	----	2.5
	Total relative amount, %			96.8	98.2	100	96.6	94.3	96.0	92.8	99.9

INF—inflorescence, L—leaves, FR—fruits; * tentatively identified; tr—traces; BP—base peak ion.

**Table 2:** Results of the different biological tests applied to the leaf essential oil (EO).

^†^ Cytotoxicity, µg/mL *				
		HC_50_	LC_50_	
		Erythrocytes	Lymphocytes	Hep-2 line
Positive controls		100 ± 0% (1,000 µg/mL)	99 ± 1% (7.5 µg/mL)	96.0 ± 0.7% (0.1/1 µg/mL)
EO		115 ± 3	71 ± 4	33 ± 2
^‡^Antibacterial susceptibility-ϕ inhibition zone, mm *				
		*S. aureus*	*E. coli*	*L. monocytogenes*
Positive control		18.2 ± 0.2 (30 µg)	18.16 ± 0.01 (4 µg)	16.55 ± 0.07 (8 µg)
EO	16 µg	97 ± 4	77 ± 4	48 ± 3
	8 µg	32 ± 2	30 ± 2	25 ± 1
	4 µg	22.5 ± 0.4	23 ± 2	17 ± 1
**Insecticidal/Repellency capacity**				
		^†^ AChE, µg/mL *	^‡^ Repellency, % *-1 µg/cm^2^	
		IC_50_	2 h	4 h
Positive controls		0.59 ± 0.02	58 ± 5	58 ± 5
EO		13 ± 1	20 ± 0	37 ± 6
**^¥^ABTS^+•^ radical-cation reactivity—TAA, mmol Trolox^®^/kg**				
BHA		2157 ± 63	EO	2249 ± 130

* Average ± standard deviation; LC_50_: 50% lethal concentration; *S. aureus*: *Staphylococcus aureus*; *E. coli: Escherichia coli*; *L. monocytogenes*: *Listeria monocytogenes*; AChE: acetylcholinesterase enzyme; TAA: total activity/capacity; ^†^ significance (*p* < 0.05, F < 0.05); ^‡^ significance (*p* < 0.05, F > 0.05, F > F_crit_); ^¥^ no significance (*p* > 0.05, F > 0.05).

## Data Availability

The data presented in this study are available on request from the corresponding author.
